# Silicon Confers Soybean Resistance to Salinity Stress Through Regulation of Reactive Oxygen and Reactive Nitrogen Species

**DOI:** 10.3389/fpls.2019.01725

**Published:** 2020-02-13

**Authors:** Yong Suk Chung, Ki-Seung Kim, Muhammad Hamayun, Yoonha Kim

**Affiliations:** ^1^Department of Plant Resources and Environment, Jeju National University, Jeju, South Korea; ^2^FarmHannong, Ltd., Daejeon, South Korea; ^3^Department of Botany, Abdul Wali Khan University, Mardan, Pakistan; ^4^School of Applied Life Science, Kyungpook National University, Daegu, South Korea

**Keywords:** canopy temperature, chlorophyll content, carboxylation efficiency, water use efficiency, nitric oxide (NO) scavenger

## Abstract

Salt stress is one of the major abiotic stressors that causes huge losses to the agricultural industry worldwide. Different strategies have been adopted over time to mitigate the negative impact of salt stress on plants and reclaim salt-affected lands. In the current study, we used silicon (Si) as a tool for salinity alleviation in soybean and investigated the influence of exogenous Si application on the regulation of reactive oxygen and reactive nitrogen species and other salt stress–related parameters of the treated plants. Our results revealed that the canopy temperature was much higher in sole NaCl–treated plants but declined in Si + NaCl–treated plants. Furthermore, the chlorophyll contents decreased with sole NaCl treatment, whereas Si + NaCl–treated plants showed improved chlorophyll contents. In addition, Si application normalized the photosynthetic responses, such as transpiration rate (*E*) and net photosynthesis rate (*P_N_*) in salt-treated plants, and reduced the activity of ascorbate peroxidase and glutathione under salt stress. The expression levels of antioxidant-related genes *GmCAT1*, *GmCAT2*, and *GmAPX1* started to decline at 12 h after addition of Si to NaCl-treated plants. Similarly, the *S*-nitrosothiol and nitric oxide (NO)–related genes were upregulated in the salt stress condition but reduced after Si supplementation. Si application downregulated genes associated with reactive oxygen species and reactive nitrogen species and reduced enzymatic and non-enzymatic antioxidants of the treated plants. Results of the current study conclude that Si mitigated the adverse effects of NaCl-induced stress by modulating the crosstalk between antioxidants and NO scavengers. It is suggested that Si may be used in agricultural systems for alleviating salt stress.

## Introduction

Soil salinity is known as a major limiting factor in crop productivity throughout the world. Machado and Serralheiro (2017) reported that 20% of irrigated lands are salt-affected globally. Plants grown under salinity stress show common symptoms or physiological responses due to the accumulation of toxic levels of ions (Na^+^, Cl^−^, and SO_2_^−^) in the cells, leading to nutritional and osmotic imbalances, and water use insufficiency (Hasegawa et al., 2000; Kim et al., 2014a; Shu et al., 2017; Rehman et al., 2019). Moreover, reactive oxygen species (ROS), such as the superoxide anion (•O_2_^−^), hydroxyl radicals (•OH^−^), and hydrogen peroxide (H_2_O_2_), accumulate in salt-stressed plants (Kim et al., 2014a; You and Chan, 2015; Jan et al., 2018; Ahmad et al., 2019). Ultimately, the plant cell walls collapse, causing the plant cells to die (Shu et al., 2017; Rehman et al., 2019). Generally, salt accumulation induces soil salinity, and it can be accelerated by irrigation of low-quality water, extensive use of chemical fertilizers, and intensive year-round cultivation (Shrivastava and Kumar, 2015). South Korea possesses limited arable land [1,691,000 hectares (ha)], and the government has been trying to expand the arable land *via* reclamation. Since the Saemangeum reclamation project was launched in 2010, a total of 29,100 ha of land was reclaimed (Choi et al., 2015). Thus, several crops, such as rice, wheat, and soybean, have been cultivated on Saemangeum reclamation land since 2010 (Choi et al., 2015).

Due to its nutritional value, soybean is one of three major worldwide crops, along with rice and wheat (Bellaloui et al., 2013; Waqas et al., 2014). Soybean is an important crop in South Korea and is a major ingredient of soy sauce and fermented soybean paste (He and Chen, 2013). Korea has a long history of soybean cultivation, and many scientists believe that soybean originated in East Asia, including South Korea, Japan, and China (Lee et al., 2011). Soybean growth and productivity are adversely affected by salt stress. Several studies conducted on the mechanism of salinity tolerance have reported several key genes related to salt stress in soybean. Although these reports have enhanced our understanding of the genetic and physiological responses of soybean to salinity stress, cultivation technologies aimed at mitigating salinity stress in soybean have shown little success despite numerous reported mechanisms (Kim et al., 2015; Liu et al., 2016; Shu et al., 2017). The environmental changes triggered by increased carbon dioxide (CO_2_) emissions have dramatically affected the cultivation of crops, leading to unexpected drought, flooding, and thermal stresses, and caused enormous yield losses (Li et al., 2016; Kim et al., 2017; Jang et al., 2018).

Silicon (Si) is the second most abundant element in soil after nitrogen, and it exists as silica (SiO_2_), silicic acid (H_4_SiO_4_), and silicate (xM^1^_2_OySiO_2_) (Epstein, 1999; Ma and Takahashi, 2002; Kim et al., 2016). Among several Si forms, plant roots usually absorb Si as H_4_SiO_4_ from the soil. The absorbed Si is then transported to the stele region, which involves the expression of two genes, named low silicon gene 1 (*Lsi1*) and low silicon gene 2 (*Lsi*2) (Ma et al., 2006; Mitani et al., 2009; Yamaji et al., 2012). Si induces various physiological responses, such as regulation of ascorbate peroxidase (APX), catalase (CAT), and glutathione (GSH) (Shi et al., 2014; Tripathi et al., 2015; Kim et al., 2016; Rizwan et al., 2019), modulation of mineral uptake (Jang et al., 2018), reinforcement of cell wall strength (Kim et al., 2016), and formation of a silica layer (Deshmukh and Bélanger, 2016). Furthermore, it is well-established that Si significantly regulates the antioxidant enzymes (CAT, APX, and GSH) to mitigate abiotic stressors, by reducing the generation of ROS (Abdel Latef and Tran, 2016; Ahmad et al., 2019).

Nitric oxide (NO) is a signaling hormone that induces various physiological processes in animals and plants (Domingos et al., 2015). NO regulates several genes involved in plant development, immunity, and stress responses (Kwon et al., 2012; Astier et al., 2018; Kim et al., 2018). It reportedly interacts with ROS as a molecular messenger to mitigate abiotic stressors (Mur et al., 2013; Astier et al., 2018). Despite many lines of research conducted on ROS and NO, the crosstalk between ROS and NO, the role of ROS and NO in mitigating salinity stress, and the role of Si treatment remain unclear. The current study was designed to elucidate the mechanism(s) responsible for the physiological changes in plants under salt stress by Si supplementation, with a particular focus on exploring the crosstalk between ROS- and NO-related enzymes and genes.

## Materials and Methods

### Plant Material and Experimental Design

We used soybean cv. Daewon, which is widely cultivated in South Korea due to its bigger seed size and higher seed weight relative to other soybean cultivars (Lee et al., 2015). Seeds of cv. Daewon were procured from the National Institute of Crop Science, Rural Development Administration of the Republic of Korea. The seeds were surface-sterilized with 70% ethanol and then rinsed with double-distilled water before sowing.

Seeds were sown into plastic trays (540 × 280 × 65 mm, with 50 holes; Shandong Co. China) provided with horticultural soil (Tobirang, Baekkwang Fertility, South Korea). The trays were transferred to the growth chamber (KGC-175 VH, Koencon, South Korea) and kept for 2 weeks under controlled conditions [07:00–21:00 h (30°C)/21:00–07:00 h (24°C), light intensity 1000 μmol m^−2^ s^−1^]. When soybean seedlings reached the vegetative stage VC (cotyledon stage), we transferred the uniform-sized soybean seedlings to a container box (380 × 490 × 80 mm) comprising double-distilled water for adaptation (2 days). To prevent anoxia or hypoxia, we supplied oxygen by an air pump during soybean cultivation (**Supplementary Figure S1**). We replaced the double-distilled water with Hoagland's solution (Hoagland salt medium, MBcell, South Korea) after the adaptation and then applied 2.0 mM Si and 100 mM NaCl once. We used 16-day-old soybean seedlings for data collection. For the determination of gene expression, we harvested plant samples after 3 to 12 h of stress exposure, while plant samples were harvested at 24 h after stress treatment for measuring the antioxidant activity and *S*-nitrosothiol (SNO) levels. Our experiment comprised the following four different treatments: (i) control, (ii) 2.0 mM Si, (iii) 100 mM NaCl, and (iv) 2.0 mM Si + 100 mM NaCl. Each treatment comprised three replications, and each replication involved 10 soybean plants (*n* = 10). The concentrations of Si and NaCl applied were verified through the screening tests and previous studies (Lee et al., 2010; Kim et al., 2014a).

### Measurement of Canopy Temperature

We measured the canopy temperature using a portable infrared camera (E5, FLIR, USA). We took the infrared images from 50–60 cm above the top of soybean canopy, and then the canopy temperature was calculated as an average temperature of the selected 20 spots on soybean leaves (**Supplementary Figure S2**). The canopy temperature images were also collected in triplicate.

### Measurement of Chlorophyll and Photosynthesis

The chlorophyll contents of leaves were measured using a portable chlorophyll meter (CCM-300; Opti-Sciences, USA). The data represent three replications (*n* = 10). For photosynthesis, we selected the second trifoliate leaf (fully expanded) from the top and measured the photosynthetic rates using a portable CO_2_ gas analyzer (LCi, ADC BioScientific Ltd., Hoddesdon, UK). After placing a clip-on LCi chamber on the leaf, we waited for 2 min (equipment acclimation) and then measured photosynthesis-related attributes. Air was continuously passed through the leaf chamber to supply CO_2_ at a proper concentration for the analysis. The concentration of CO_2_ in the chamber was approximately 370 ± 7 μmol mol^–1^, and the water vapor pressure in the leaf chamber was maintained at around 2.10 ± 0.23 kPa. Net photosynthetic rate (*P_N_*), stomatal conductance (*g_s_*), transpiration rate (*E*), and internal concentration of CO_2_ (*C_i_*) were measured. Finally, the values of the instantaneous carboxylation efficiency (*P_N_*/*C_i_*) and instantaneous water use efficiency (*P_N_*/*E*) were calculated from the abovementioned values. Data were replicated thrice (*n* = 10).

### Antioxidant Activity

Fresh leaf samples were used to analyze APX, CAT, and GSH activities. An Ascorbate Peroxidase Microplate Assay Kit (Cohesion Biosciences, UK) was used to quantify the APX activity in soybean. A 100 mg sample of leaf tissues was homogenized in 1 ml of assay buffer on ice and then centrifuged at 13,000*g* at 4°C for 20 min. The supernatant used for detection was kept on ice throughout the experiment. APX activity was quantified according to the manufacturer's protocol. CAT activity was analyzed using the CAT Assay Kit (Amplex Red Catalase Assay Kit, Invitrogen, USA) as per the manufacturer's procedure. First, we prepared a 10 mM stock solution of Amplex Red reagent, 1× working solution of reaction buffer, 100 U ml^–1^ solution of horseradish peroxidase, 20 mM H_2_O_2_ working solution, and 1,000 U ml^–1^ CAT solution. Serial dilution of CAT (0–4.0 U ml^–1^) was done in 1× reaction buffer, and the buffer without CAT was used as a negative control. Exactly 25 µl of diluted samples and controls were poured into the wells of a 96-well microplate, followed by 25 µl of 40 µM H_2_O_2_ solution to each microplate well containing the samples, and then the reaction mixture was left at room temperature for 30 min. The second step of the reaction was conducted by adding 50 µl of the Amplex Red/Horseradish peroxidase (HRP) working solution to each microplate well. The plate was incubated at 37°C for 30 min. Finally, the absorbance was measured in a microplate reader at an excitation wavelength range of 530–560 nm and emission wavelength of 590 or 560 nm. GSH activity was measured using a GSH Assay Kit (Cohesion Biosciences). A 100 mg aliquot of fresh samples was homogenized with 1 ml of assay buffer on ice and then centrifuged at 8,000*g*, 4°C for 10 min. The supernatant was transferred to an empty centrifuge tube and kept on ice until detection. All reagents were warmed to 37°C before use and then added to the microplate. After that, we followed the manufacturer's procedure. GSH activity was measured at 412 nm. The antioxidant activity was repeated three times (*n* = 3).

### Determination of SNO Content

To analyze the SNO content in soybean, we followed the protocol of Yun et al. (2011). A 100 mg aliquot of fresh leaf sample was used for SNO analysis. The leaf samples were ground to a fine powder using a mortar and pestle with liquid nitrogen. Then, 1 ml of extraction buffer (1× PBS, pH 7.4) was added to the powder and centrifuged at 12,000*g* for 10 min. After centrifugation, the supernatant was placed in an empty tube and centrifuged at 13,000*g* for 10 min. To quantify the protein, 1.5 ml of dye reagent was added to 30 μl of the extracted protein, and the assay was performed using the Pierce Coomassie Protein Assay Kit (Thermo Fisher Scientific, USA). After incubation at room temperature for 10 min, readings were recorded using a spectrophotometer at 595 nm. To measure the SNO content, 100 μl of the plant extract was injected into the reaction vessel of the Sievers' Nitric Oxide Analyzer (NOA 280i, GE Water & Process Technologies, Ratingen, Germany) containing the reducing agent, including CuCl/cysteine with water.

### Quantitative Real-Time PCR

Total RNA was isolated from fresh soybean leaf tissues using TRIzol reagent (Invitrogen, USA) to analyze the influence of specific genes related to antioxidant activity. Using liquid nitrogen, fresh soybean leaf samples were finely ground, and then 1 ml of TRIzol reagent was added immediately. Samples were centrifuged at 13,000 rpm, 4°C for 5 min. The supernatant was transferred to an empty 1.7 ml tube, and chloroform and isopropanol were added for phase separation and RNA precipitation, respectively. Centrifuge steps were conducted in between the additions mentioned above. Isolated RNA pellets were washed with 75% Diethyl pyrocarbonate (DEPC)-treated ethanol, dissolved in RNase-free water, and treated with DNaseI. Total RNA was used for complementary DNA (cDNA) synthesis by following the manufacturer's protocol (cDNA Synthesis Kit, Phile, Korea). The cDNA was used as a template for real-time PCR (Eco™ Real-Time PCR, Illumina, USA). During the real-time PCR process, 2× QuantiSpeed SYBR Mix (Phile, Korea) was used as the reaction mixture, and PCR was conducted according to the manufacturer's protocol. *GmUBI* was used as the reference gene for data normalization. The experiment was replicated three times. The primers used are listed in **Supplementary Table S1**.

### Statistical Analysis

The experiment was repeated three times, and each replicate comprised 10 plants. The data collected from each experiment were pooled, analyzed statistically, and then subjected to Duncan's multiple range test to identify the significant differences among the treatments, using the statistical software SAS (version 9.2; SAS Institute, Inc., Cary, NC, USA).

## Results

### Influence of Canopy Temperature and Chlorophyll Content

**Figure 1:**
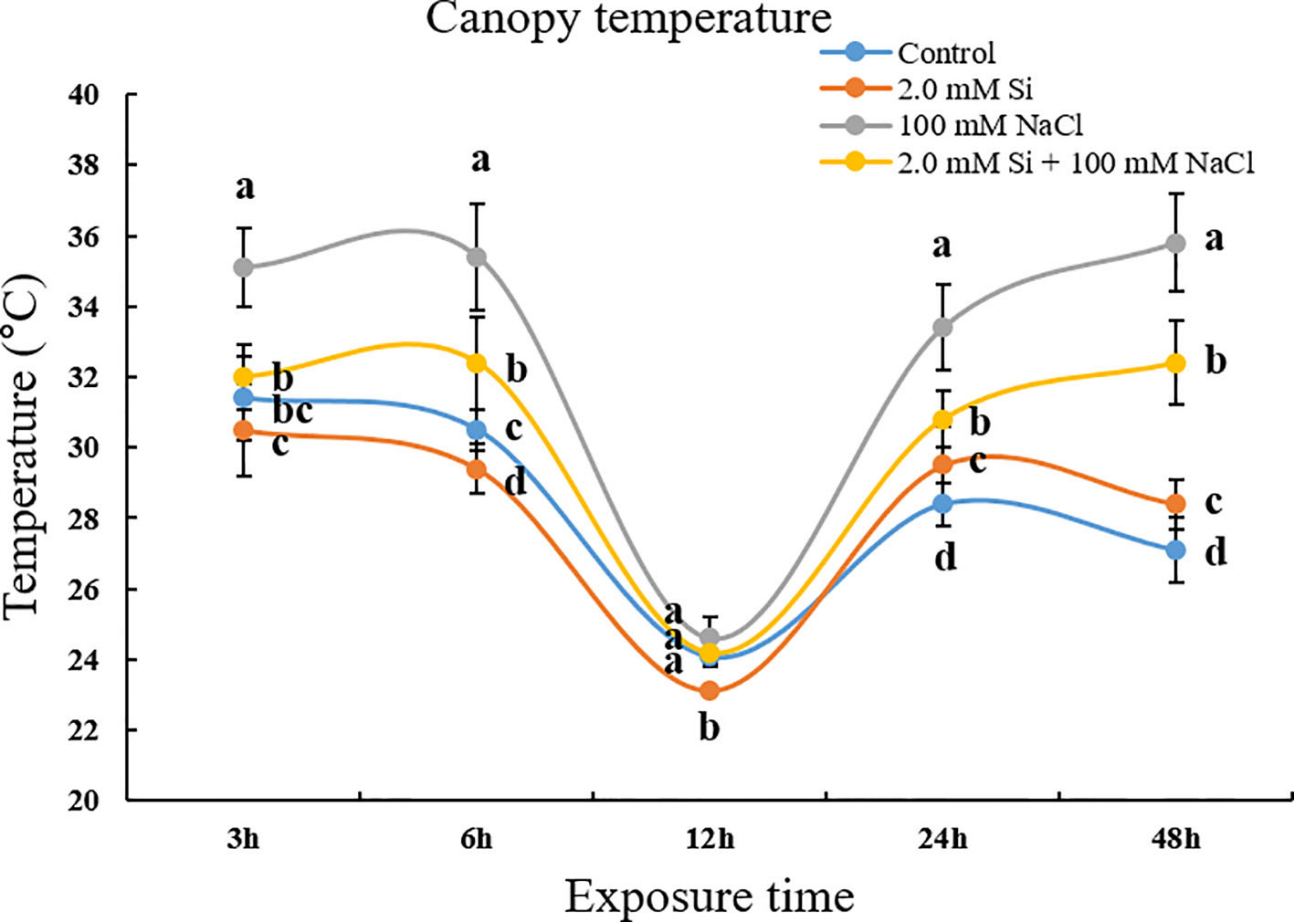
Canopy temperature after single or combined Si and NaCl treatments in soybean plants. Data were collected at 3, 6, 12, 24, and 48 h after stress exposure. Temperature data were calculated by an average temperature of the selected 20 spots on soybean leaves per treatment. In the figure, error bars denote standard deviation, and different letters near error bars indicate significant differences by Duncan's multiple range test (*P* < 0.05). Data were collected in triplicate and are presented as the average ± standard error (*n* = 10).

**Figure 2:**
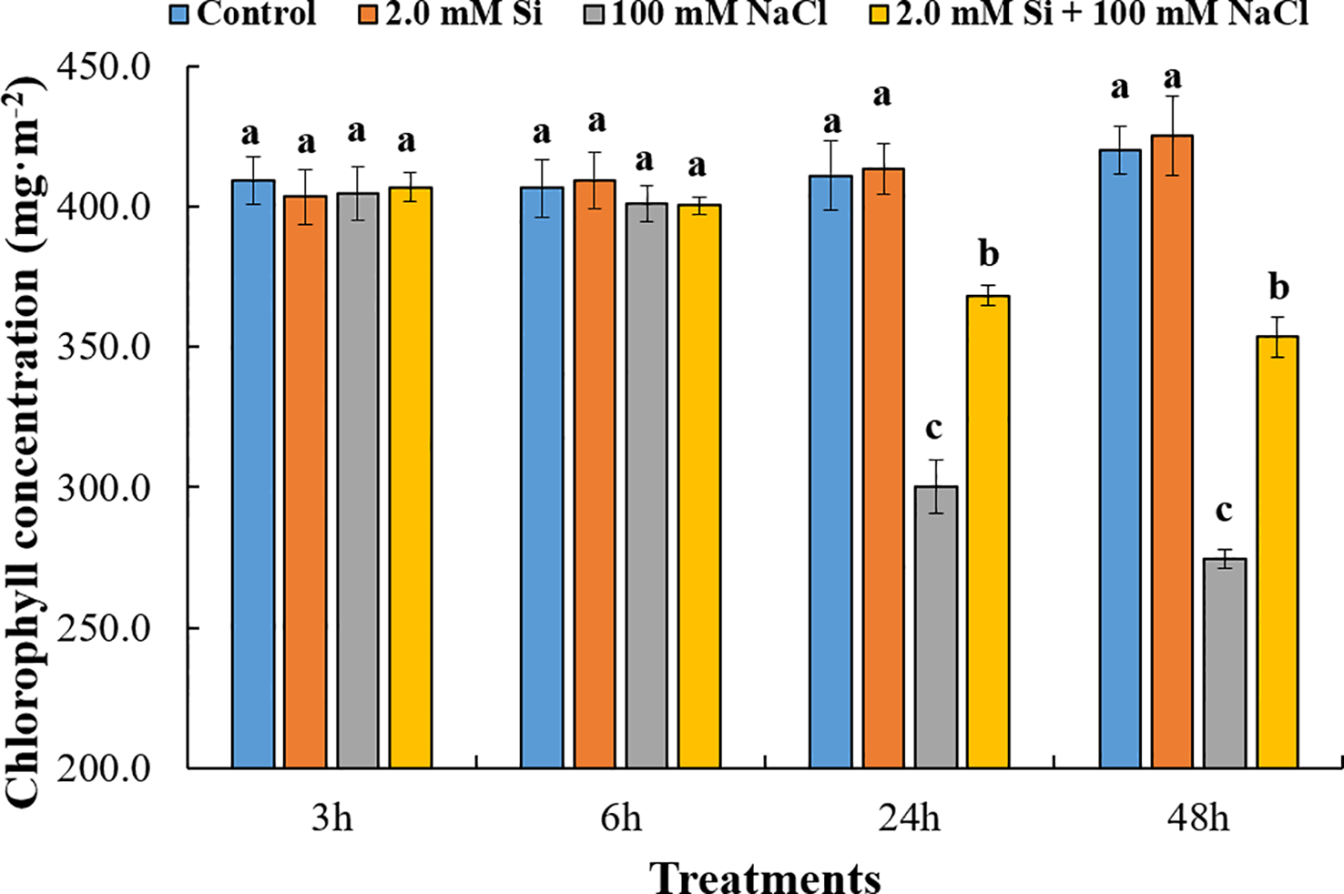
Influence of chlorophyll concentration after single or combined Si and NaCl treatments in soybean plants. Data were collected at 3, 6, 24, and 48 h after stress exposure. Different letters above error bars indicate significant differences by Duncan's multiple range test (*P* < 0.05). Data were collected in triplicate and are presented as the average ± standard error (*n* = 10).

To evaluate the transpiration efficiency in single or combined Si and NaCl treatments, we measured canopy temperature using a portable infrared camera. After briefly exposing the soybean plants to salt stress (3 h), the canopy temperature of NaCl-treated soybean plants was significantly different from that of plants subjected to other treatments (**Figure 1**). At all the time points, soybean plants treated with sole NaCl showed a higher temperature (35°C) than plants subjected to other treatments (30–32°C). Conversely, soybean plants treated with Si + NaCl showed a reduced canopy temperature relative to those treated with sole NaCl (**Figure 1**). The chlorophyll content did not vary significantly among the various treatments at 3–6 h after stress exposure, but was dramatically reduced at 24–48 h after stress exposure (**Figure 2**). The chlorophyll contents declined by 27.3% and 10.9% in sole NaCl and Si + NaCl treatments, respectively, after stress exposure for 24 h. A similar pattern was observed for 48 h stress exposure, as the chlorophyll contents decreased by 34.7% in sole NaCl and 15.9% in Si + NaCl treatments, respectively, as compared with the control (**Figure 2**). Although the chlorophyll contents decreased significantly in response to Si + NaCl and sole NaCl treatments, the decline in chlorophyll was much higher in the plants exposed to sole NaCl when compared with the control (**Figure 2**).

### Analysis of Photosynthesis

**Figure 3:**
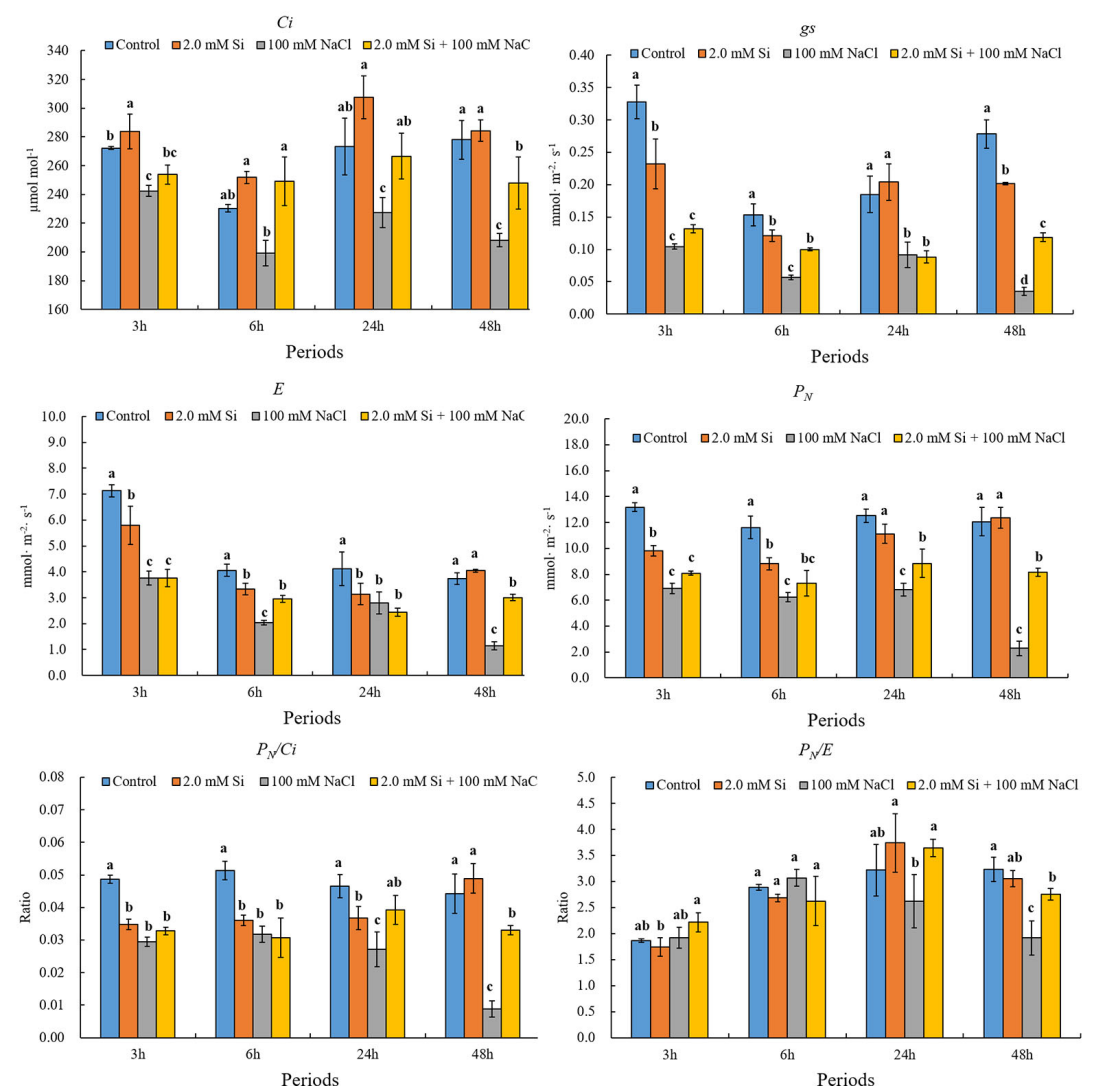
Effect of single or combined Si and NaCl treatment on net photosynthesis (*P_N_*), transpiration rate (*E*), stomatal conductance (*g_s_*), intercellular CO_2_ concentration (*C_i_*), *P_N_*/*C_i_*, and *P_N_*/*E* in soybean plants. Data were collected at 3, 6, 24, and 48 h after stress exposure. Different letters above error bars indicate significant differences by Duncan's multiple range test (*P* < 0.05). Data were collected in triplicate and are presented as the average ± standard error (*n* = 10).

Based on the results of analysis of variance (ANOVA), all photosynthetic traits shows significant difference (**Table 1**). According to our results, the net photosynthesis (*P_N_*) of the control plants was decreased dramatically by 46.4% (6 h) to 81.1% (48 h) in sole NaCl–treated plants (**Figure 3**). The same pattern was detected at all the time points. Overall, the transpiration rate (*E*) was reduced in salt stress conditions, but less so by Si + NaCl treatment (declines of 27.1% in 6 h and 19.5% in 48 h) than sole NaCl treatments (declines of 49.8% in 6 h and 69.3% in 48 h) irrespective of the duration of stress exposure (**Figure 3**). The stomatal conductance (*g_s_*) showed the same tendency as *E*, at all periods (**Figure 3**). Furthermore, the intercellular CO_2_ concentration (*C_i_*) of the control plants was reduced dramatically, by approximately 2.2% (24 h) and 10.8% (48 h) with sole NaCl treatment (see **Figure 3**).

**Table 1:** Analysis of variance (ANOVA) results for the effect of treatments (Tre), period (Per), and their interaction on stomatal conductance (*g_s_*), net photosynthetic rate (*P_N_*), transpiration rate (*E*), internal concentration of CO_2_ (*C_i_*), instantaneous water use efficiency (*P_N_*/*E*), and instantaneous carboxylation efficiency (*P*_N_/*C_i_*).

Trait	Source of variation	*df*	Mean square	*F*-value	*P*-value
*g_s_*	Tre	3	0.199794	115.94	<.0001
	Per	3	0.051798	30.06	<.0001
	Rep (Tre)	2	0.002838	1.65	0.1967
	Tre × Per	9	0.013323	7.73	<.0001
*P_N_*	Tre	3	314.2517	133.08	<.0001
	Per	3	14.32285	6.07	0.0007
	Rep (Tre)	2	8.917108	3.78	0.0255
	Tre × Per	9	19.56759	8.29	<.0001
*E*	Tre	3	38.88273	70.14	<.0001
	Per	3	37.47944	67.61	<.0001
	Rep (Tre)	2	0.196233	0.35	0.7026
	Tre × Per	9	3.973437	7.17	<.0001
*C_i_*	Tre	3	7808.454	7.54	0.0001
	Per	3	9667.917	9.33	<.0001
	Rep (Tre)	2	2069.996	2	0.1399
	Tre × Per	9	4044.862	3.9	0.0002
*P*_N_/*E*	Tre	3	1.598927	4.5	0.0049
	Per	3	11.55325	32.52	<.0001
	Rep (Tre)	2	0.321394	0.9	0.4073
	Tre × Per	9	1.502979	4.23	<.0001
*P_N_*/*C_i_*	Tre	3	0.003453	49.09	<.0001
	Per	3	0.000111	1.57	0.1995
	Rep (Tre)	2	0.00021	2.99	0.0538
	Tre × Per	9	0.00049	6.96	<.0001

In the table, “Rep (Tre)” and “Tre × Per” indicate replication within treatment and interaction between treatments and periods, respectively. Data represent triplicate measurements.

According to our results, *P_N_*/*C_i_* showed the same pattern as *P_N_*, whereas *P_N_*/*E* showed a different trend (**Figure 3**). In response to short stress exposure (3 and 6 h), *P_N_*/*E* did not decrease in NaCl treatments but did decrease significantly in sole NaCl–treated plants exposed to 24 and 48 h of stress as compared with the control (**Figure 3**). Conversely, Si + NaCl–treated soybean plants showed better *P_N_*/*E* than sole NaCl–treated plants. As a result, *P_N_*/*E* showed a similar pattern as observed for *P_N_*, *E*, and *g_s_* during the 48 h of salt stress.

### Antioxidant Activity and Its Expression Level Under Salinity Stress Condition

**Figure 4:**
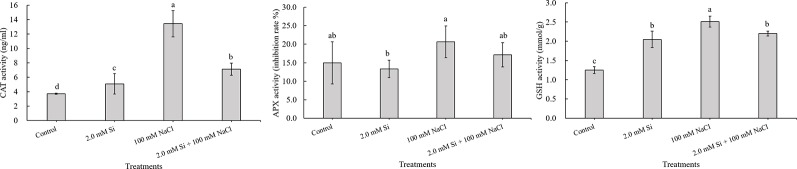
Changes in antioxidant activity after single or combined Si and NaCl. Data were collected after salt stress for 24 h. Different letters above error bars indicate significant differences by Duncan's multiple range test (*P* < 0.05). Data were collected in triplicate and are presented as the average ± standard error (*n* = 3).

The CAT activity was 3.6-fold higher in sole NaCl–treated plants and 1.9-fold higher in CAT activity for Si + NaCl–treated plants relative to the control. Thus, the enhanced activity of CAT induced by NaCl was mitigated by the addition of Si (**Figure 4**). The activity of APX was also 1.4-fold higher in sole NaCl–treated plants than in control plants, whereas a marginal 1.7% decrease in APX activity was noted in Si + NaCl–treated plants than sole NaCl–treated plants (**Figure 4**). GSH activity displayed a similar trend to the CAT activity. Maximum GSH (2-fold higher than control plant) was detected in sole NaCl–treated plants, but Si + NaCl treatment showed 12.4% less GSH activity than the sole NaCl–treated plants (see **Figure 4**).

**Figure 5:**
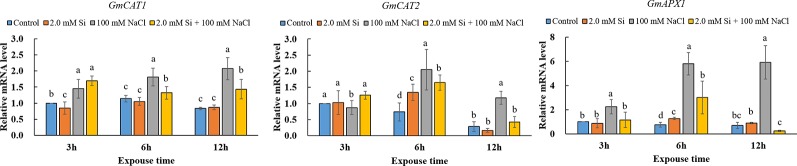
Relative messenger mRNA expression level of antioxidant-related genes (*GmCAT1*, *GmCAT2*, *GmAPX1*) after single or combined Si and NaCl. Data were collected at 3, 6, and 12 h after stress exposure. Different letters above error bars indicate significant differences by Duncan's multiple range test (*P* < 0.05). Data were collected in triplicate and are presented as the average ± standard error (*n* = 3).

Our results revealed that the activity of ROS scavengers, such as CAT, APX, and GSH, dramatically increased in salt stress conditions, but the effect of salt stress was alleviated by Si application. To confirm the above tendency at the genetic level, we analyzed the expression level of antioxidant-related genes, such as *GmCAT*s and *GmAPX1*. Interestingly, the expression level of *GmCAT1, GmCAT2*, and *GmAPX1*, respectively, was upregulated 1.6- to 2.5-fold, 2.8- to 4.2-fold, and 7.8- to 8.6-fold, respectively, in NaCl-treated soybean plants relative to the control plants after 6 h of stress exposure. Conversely, during 6 and 12 h of exposure, the expression level of *GmCAT1, GmCAT2*, and *GmAPX1* in Si + NaCl–treated soybean plants were, respectively, downregulated 27–30.4%, 19.5–64.4%, and 48–95.8% as compared with sole NaCl–treated plants (**Figure 5**). In all cases, the expression level of these genes with Si + NaCl and sole NaCl treatments increased with an increase in the duration of stress exposure. However, the expression level of antioxidant-related genes was lowered in Si + NaCl–treated plants relative to the sole NaCl–treated plants (**Figure 5**).

### Effect of Si Application on the Changes of SNO and Its Related Genes

**Figure 6:**
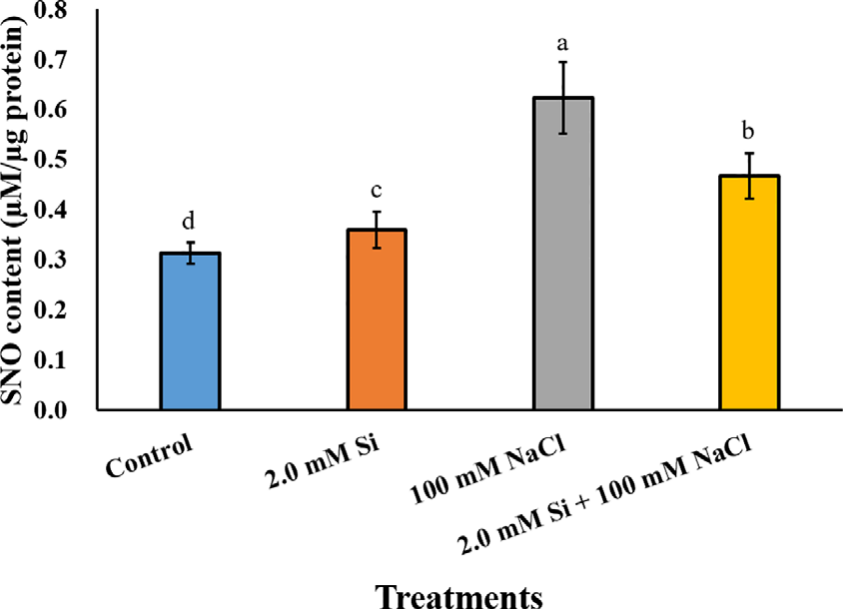
*S*-nitrosothiol (SNO) content after single or combined Si and NaCl. Data were collected after salt stress for 24 h. Different letters above error bars indicate significant differences by Duncan's multiple range test (*P* < 0.05). Data were collected in triplicate and are presented as the average ± standard error (*n* = 3).

**Figure 7:**
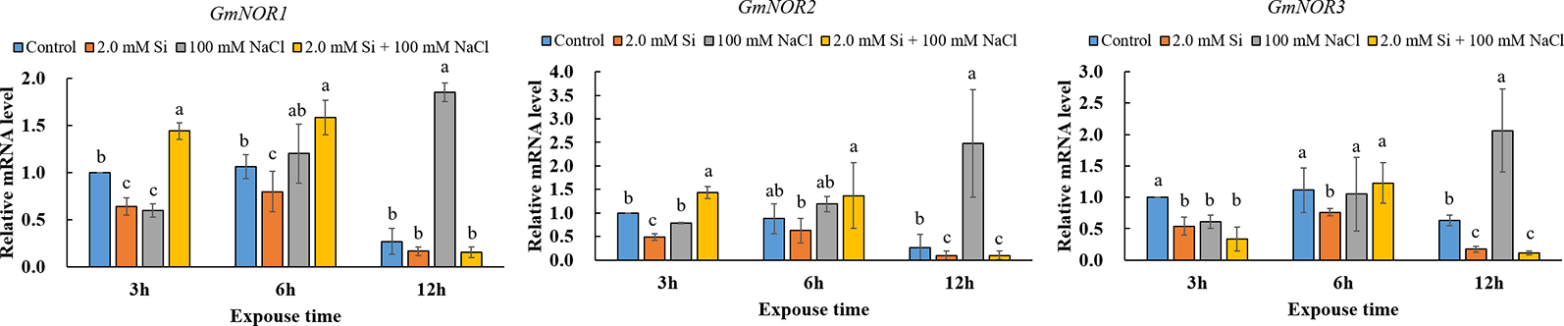
Relative mRNA expression level of SNO-related genes (*GmNOR1*, *GmNOR2*, *GmNOR3*) after single or combined Si and NaCl. Data were collected at 3, 6, and 12 h after stress exposure. Different letters above error bars indicate significant differences by Duncan's multiple range test (*P* < 0.05). Data were collected in triplicate and are presented as the average ± standard error (*n* = 3).

The SNO content increased significantly in all Si + NaCl–treated plants when compared with the control, and was highest in the sole NaCl–treated soybean (2-fold higher than the control). Conversely, the SNO content was reduced by 24.2% in Si + NaCl–treated plants relative to sole NaCl–treated plants (**Figure 6**). To confirm the SNO result, we measured the messenger RNA (mRNA) expression levels of SNO-related genes, such as *GmNOR1*, *GmNOR2*, and *GmNOR3*, at 3, 6, and 12 h of salt stress. In the first 3 h of stress, all SNO-related genes were downregulated by 21% to 67% in sole NaCl–treated plants in comparison to the control plants (**Figure 7**). However, toward the end of 6 h exposure, each SNO-related gene showed either a slight increase in sole NaCl–treated plants (*GmNOR1*: 12.1%, *GmNOR2*: 35.2%) or the same expression level (*GmNOR3*: 9.8%) as the control plants (**Figure 7**). By the end of 12 h stress exposure, the expression of all three genes exhibited a dramatic increase of 2- to 3-fold in sole NaCl–treated plants over the other treatments (**Figure 7**). The expression level of all SNO-related genes was downregulated with sole Si treatment, and the same pattern was observed at all the time points (**Figure 7**).

## Discussion

Plants grown in agricultural systems are exposed to many environmental stresses that limit their growth. Plant growth is usually due to the improvement of plant resistance to abiotic stressors, such as salinity (Abdel Latef et al., 2019a), and there is ample evidence that Si plays a favorable role in plant growth, mineral nutrition, mechanical strength, and resistance to fungal diseases (Hamayun et al., 2010a; Jan et al., 2018; Rizwan et al., 2019). When the sodium ion (Na^+^) and chloride ion (Cl^−^) are present in soil at higher than normal concentrations, plants suffer from the adverse effects of NaCl-induced salt stress (Tavakkoli et al., 2010). Salt stress causes nutritional imbalances, such as ion toxicity, water-potential degradation, and compromised uptake and transport of ions (Ahmad et al., 2019; Ali et al., 2019). Salt stress also causes growth inhibition in all plants because it affects all processes necessary for a plant to survive, such as growth, photosynthesis, energy metabolism, and protein synthesis (Parida and Das, 2005; Jan et al., 2018; Ahmad et al., 2019). Accumulation of Na^+^ and Cl^−^ in the plant cell accelerates programmed cell death due to the lipid peroxidation of cellular membranes caused by an imbalance of ions (Abdel Latef et al., 2017; Abdel Latef et al., 2019b; Khan et al., 2019).

Plants are sessile organisms, and so have developed a self-defense system to cope with abiotic and biotic stress conditions (Zhu, 2016). In response to heat, plants transpire to reduce their internal temperature (Bita and Gerats, 2013). The stomata play a key role in both transpiration and photosynthesis, responding to humidity and total gas exchange. The opening of the stomata facilitates the removal of water vapor from the plant leaf to the atmosphere (Yonemura et al., 2019), which reduces the plant temperature. For the above reasons, the canopy temperature measurement of plants is broadly used for estimation of photosynthetic efficiency (Furbank et al., 2019). According to our study, NaCl-treated soybean plants showed a higher temperature (35°C) than the other plants. Moreover, both decreased transpiration and net photosynthesis were recorded for sole NaCl–treated soybean plants. However, the temperature was lower in Si + NaCl–treated plants, while transpiration and net photosynthesis rate were comparatively higher in Si + NaCl–treated plants. It suggests that Si application mitigated the effect of salt stress by regulating the stomatal conductance and rate of transpiration. When plants encounter salinity stress, the stomata close to minimize uptake of Na^+^ and Cl^−^ from soil (Hanin et al., 2016; Kim et al., 2018). Therefore, our results confirm the hypothesis that soybean plants recognize salinity stress at 100 mM NaCl treatment and, consequently, close the stomata to hinder the accumulation of Na^+^ and Cl^−^ in the plant cells. Hence, canopy temperature increased while net photosynthesis decreased in sole NaCl–treated soybean. On the contrary, decreased canopy temperature and ameliorated transpiration and net photosynthesis occurred in salt-stressed soybean plants when supplemented with Si in the growth medium, which may be due to the fact that Si improves photosynthetic rate, which is associated with leaf ultrastructure, chlorophyll content, and ribulose biphosphate carboxylase activity (Hamayun et al., 2010b). Similar results were also documented in pea and rice (Kim et al., 2014b; Tripathi et al., 2015).

Chlorophyll is an important pigment for photon harvest in the photosystem II (PSII) and photosystem I (PSI) during photosynthesis (Caffarri et al., 2014). Thus, change of leaf color and, especially, monitoring of leaf chlorophyll contents are widely used as an indicator of plant status under abiotic stress conditions (Abdel Latef et al., 2019b). According to our results, chlorophyll content declined significantly in Si + NaCl–treated and sole NaCl–treated plants, particularly. The chloroplast is one of the main photosynthesis apparatus, as PSII and PSI occur in the chloroplast (Caffarri et al., 2014). If plants encounter extreme stress, ROS is accumulated in the various cell organelles, such as chloroplast, mitochondria, and endoplasmic reticulum, which could ultimately destroy the cellular membrane and the cell organelle (Broetto et al., 2007; Caffarri et al., 2014; Kim et al., 2018). For this reason, the sole NaCl–treated plants showed a significant decrease in chlorophyll contents, but this was ameliorated by the addition of Si in the growth medium. Our current findings are in agreement with previous reports that silicate partially offsets the negative impact of NaCl stress and increases the tolerance of plants to NaCl salinity by raising the antioxidant activity, such as CAT, APX, and GSH (Hamayun et al., 2010a; Zhu et al., 2019). In plants, Si is absorbed from the soil and then transported from roots to shoots *via* the transpiration stream (Gilliham et al., 2011). When Si reaches the leaves, it produces a silica body and silica cell to confer physical strength against abiotic and biotic stressors (Kim et al., 2014b). Moreover, Si enhances resistance to various environmental stressors, such as salt, drought, heavy metal, and high temperature, through regulation of the antioxidant activity, as well as modulation of phytohormones (Kim et al., 2016; Kim et al., 2017; Ahmad et al., 2019; Rizwan et al., 2019).

During photosynthesis and respiration, plants continuously produce ROS, such as singlet oxygen, •O_2_^−^, H_2_O_2_, and •OH^−^, and these ROS induce oxidative damage in the cell organelle (Sharma et al., 2012; Kim et al., 2017; Abdel Latef et al., 2018). Therefore, plants must scavenge excess ROS to avoid plant cell damage (Sharma et al., 2012). Plants can scavenge ROS by regulating the non-enzymatic and enzymatic antioxidants (Wu et al., 2017). According to Racchi (2013), CAT and APX are classified as the main enzymatic antioxidants, as they participate in the decomposition of H_2_O_2_ to water. However, non-enzymatic antioxidants, which include carotenoids, tocopherols, ascorbate, and GSH, prevent lipid peroxidation and promote membrane protection (Racchi, 2013; Kim et al., 2017; Wu et al., 2017). The antioxidant GSH also participates in ROS scavenging by regulating dehydroascorbate reductase to induce the reduction of dehydroascorbate to ascorbate. Ascorbate is known as a source of APX, which scavenges oxygen from hydrogen peroxidase to form water. In this way, the activity of GSH also an important indicator of oxidative stress. Enzymatic antioxidants remove ROS under salt stress conditions, so the measurement of the antioxidants' activity is valuable in determining stress resistance (Chen et al., 2011; Rejeb et al., 2014; Ali et al., 2019). According to our results, the activity of CAT, APX, and GSH, respectively, significantly increased in sole NaCl–treated plants but was drastically reduced in Si + NaCl–treated plants. In particular, the activity of CAT and APX, respectively, was downregulated by modulation of the related genes *GmCAT1*, *GmCAT2*, and *GmAPX1*.

NO is a signal molecule in plants that regulates plant development and immunity (Hussain et al., 2016). According to Zurbriggen et al. (2010), NO can reduce disease susceptibility by ROS scavenging, as it participates in the regulation of antioxidant activity by reducing the ROS level in plants. NO is a highly reactive radical that must be converted into a non-toxic form, such as *S*-nitrosoglutathione (GSNO) and SNO (Hussain et al., 2016; Kim et al., 2018). GSNO and SNO are classified as reactive nitrogen species (RNS) because they act as NO donors or carriers in plant cells (Hussain et al., 2016). To elucidate the effect of Si on the regulation of ROS and RNS, we measured the SNO contents, as well as the expression of SNO-related genes. According to Wang et al. (2013), *S*-nitrosoglutathione reductase (*GSNOR*) is used as an indirect regulator of SNO because it controls the GSNO content (Mur et al., 2013). For this reason, we measured the mRNA expression levels of *GmNOR1, GmNOR2*, and *GmNOR3*. Our current study shows that the salt-treated plants induce a high level of mRNA activity of *GmNOR1, GmNOR2*, and *GmNOR3*, respectively. In contrast, Si + NaCl–treated plants exhibited significantly low mRNA activity, so our results imply that Si has a reasonable role in alleviating NaCl-induced salt stress through downregulation of SNO-related genes, such as *GmNOR1, GmNOR2*, and *GmNOR3*.

Photosynthesis is the most important process in plants for producing energy to complete their life cycle (Evans, 2013). Measurement of the photosynthesis-related factors, such as transpiration, stomatal conductance, and CO_2_ concentration, has been broadly used for assessing photosynthesis. The gaseous exchange accompanies transpiration and enables temperature regulation in plants. We measured the canopy temperature to predict transpiration efficiency after solo or combined Si and NaCl treatments. We found canopy temperature to be strongly related to stomal regulation. The stomata have immense control over canopy transpiration (Wohlfahrt et al., 2012), which is closely linked to carbon assimilation by leaves (Murchie et al., 2018). Accordingly, *g_s_*, *E*, and *P_N_* were much lower in the salt-treated plants than control plants. However, *g_s_*, *E*, and *P_N_* were increased in Si + NaCl–treated plants. In general, *C_i_*, *E*, and *P_N_* are used to predict carboxylation efficiency (*P_N_*/*C_i_*) and water use efficiency (*P_N_*/*E*) (Sudhir and Murthy, 2004; Hwang and Choo, 2017).

## Conclusion

According to our results, salinity stress induced a decrease in net photosynthesis and transpiration, but increased canopy temperature. We assumed that net photosynthesis reduced because of the significant decrease in chlorophyll content, as observed in sole NaCl–treated plants. Decreased chlorophyll content might be caused by chloroplast damage induced by the accumulation of ROS. Therefore, sole NaCl–treated plants showed high activity of APX, CAT, and GSH to scavenge ROS. Our result confirmed that such antioxidant activity was modulated at the genetic level (*GmNOR1, GmNOR2*, and *GmNOR3*). On the contrary, Si + NaCl–treated plants showed ameliorated phenomena across all phenotypes. According to our results, during salinity stress, the addition of Si not only downregulated ROS- and RNS-related genes but decreased the enzymatic and non-enzymatic antioxidants as compared with sole NaCl–treated plants. Thus, decreased ROS and SNO (NO donor) mitigated the adverse effect of sole salt stress on chlorophyll contents. Finally, Si + NaCl treatment improved the chlorophyll content and net photosynthesis and decreased the canopy temperature.

## Data Availability Statement

The datasets generated for this study are available on request to the corresponding author.

## Author Contributions

YC and K-SK equally wrote the manuscript and collected the data. MH analyzed the data. YK inspected the experimental design and revised the manuscript.

## Conflict of Interest

Author K-SK was employed by FarmHannong, Ltd.

The remaining authors declare that the research was conducted in the absence of any commercial or financial relationships that could be construed as a potential conflict of interest.

## Supplementary Material

Supplementary Table S1 The primers used for real-time PCR.

Supplementary Figure S1 Oxygen application method for hydroponic.

Supplementary Figure S2 Infrared image to measure canopy temperature.
