# Supplementary material for: Silicon Confers Soybean Resistance to Salinity Stress Through Regulation of Reactive Oxygen and Reactive Nitrogen Species
Source: Front Plant Sci. 2020 Feb 13;10:1725. doi: 10.3389/fpls.2019.01725 (PMC7031409; doi:10.3389/fpls.2019.01725)
Supplement: Supplementary Table S1 — The primers used for real-time PCR. [file Table_1.docx]

**Supplementary data**

**Supplementary Table S1.** The primers used for real-time PCR.

| Gene | Primer sequences |
| --- | --- |
| *GmAPX1* | Forward: AGCGCTAACAACGGTCTTGA  Reverse: GTGACCTCAACGGCAACAAC |
| *GmCAT1* | Forward: AGCTAGCGCAAAGGGTTTCT  Reverse: AAGGTTTCAGGGCTACCACG |
| *GmCAT2* | Forward: GCGCTAGTGCAAAGGGTTTC  Reverse: AAGGTTTCAGGGCTACCACG |
| *GmGSNOR1* | Forward: TAGGTGTTGCAGCATCAGGG  Reverse: TCTACAAGCCAAGGCACCTG |
| *GmGSNOR2* | Forward: TAGGTGTTGCAGCATCAGGG  Reverse: TCTACAAGCCAAGGCACCTG |
| *GmGSNOR3* | Forward: TAGGTGTTGCAGCATCAGGG  Reverse: TCTACAAGCCAAGGCACCTG |
