# Supplementary figures and images for: Silicon Confers Soybean Resistance to Salinity Stress Through Regulation of Reactive Oxygen and Reactive Nitrogen Species
Source: Front Plant Sci. 2020 Feb 13;10:1725. doi: 10.3389/fpls.2019.01725 (PMC7031409; doi:10.3389/fpls.2019.01725)

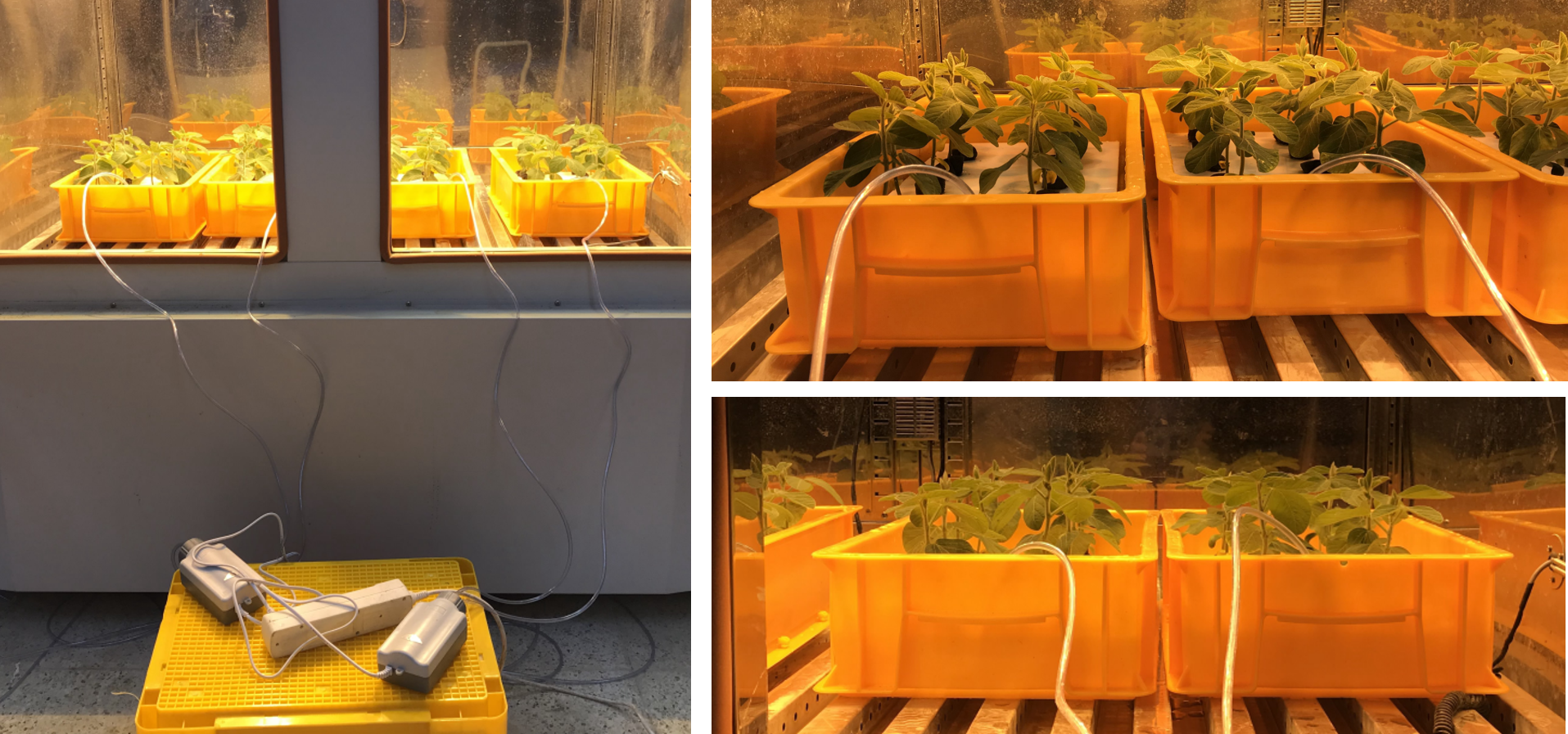

Supplement: Supplementary Figure S1 — Oxygen application method for hydroponic. [file Image_1.tif]

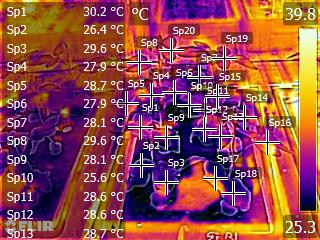

Supplement: Supplementary Figure S2 — Infrared image to measure canopy temperature. [file Image_2.tif]
